# Cerebral palsy in Moldova: subtypes, severity and associated impairments

**DOI:** 10.1186/s12887-018-1305-6

**Published:** 2018-10-19

**Authors:** Ecaterina Gincota Bufteac, Guro L. Andersen, Vik Torstein, Reidun Jahnsen

**Affiliations:** 1 Department of Health Sciences, Oslo Metropolitan University, PO box 4 St. Olavs plass, No-0130 Oslo, Norway; 2 Early Intervention Center ‘Voinicel’, Drumul Taberei str., nr.2A, Chisinau, Moldova; 3 The Cerebral Palsy Register of Norway (CPRN), Department of Paediatrics, Vestfold Hospital Trust, Tonsberg, Norway; 4 Department of Clinical and Molecular Medicine, Norwegian University of Science and Technology, PO Box 8905, 7491 Trondheim, Norway; 5 The Cerebral Palsy Follow-up Program (CPOP), Department of Clinical Neurosciences for Children, Oslo University Hospital, Box 4950, Nydalen, 0424 Oslo, Norway

**Keywords:** Cerebral palsy, Moldova, Epidemiology, Subtype, Severity

## Abstract

**Background:**

Moldova is ranked as one of the countries in Europe with the lowest income per capita and with a relatively high infant and maternal mortality rate. Information on neurodisabilities in general is limited, and regarding cerebral palsy (CP) in particular, it is completely lacking. The aim of this study was therefore to make a crude estimate of the prevalence of CP and to describe subtypes and the severity of motor impairments and associated problems in this country.

**Methods:**

Children with CP born 2009–2010, attending the National Hospital Institute of Mother and Child, the reference hospital for ~ 75% of children in Moldova with neurological disabilities, were identified from medical records.

**Results:**

Among 207 children with CP (estimated prevalence 3.4 per 1000 live births), 185 (mean age 7.3 years; 36% girls) had detailed information. Thirty seven (20%) children had spastic unilateral, 113 (61%) spastic bilateral, 22 (12%) dyskinetic and 9 (5%) children had ataxic CP. The subtype was unclassified in four children. Among all children, 93 (51%) had epilepsy, 109 (59%) intellectual disability, 42 (23%) severe vision and 10 (5%) hearing impairments while 84 (45%) children had severe speech impairments. Fifty-two (28%) children were born prematurely, and 46 (25%) had Apgar scores below 7 at five minutes.

**Conclusion:**

Compared to other European studies, the distribution of CP subtypes was different in Moldova. Moreover, the estimated prevalence, the proportions with severe motor and associated impairments and of children born at term were higher in Moldova while the proportion with low Apgar did not differ. The findings may suggest different etiological pathways causing CP in Moldova than in other European countries. A national register is warranted for quality assurance and improvement.

## Background

Cerebral palsy (CP) describes a group of permanent disorders of the development of movement and posture, causing activity limitations that are attributed to non-progressive disturbances occurring in the developing foetal or infant brain. The motor impairments are often accompanied by disturbances of sensation, perception, cognition, communication, and behaviour, by epilepsy and by secondary musculoskeletal problems [1].

Considering the significant differences in perinatal and infant mortality between high- and low-income countries, it may seem paradoxical that the prevalence of CP has been reported to be similar, at around 2–3 per 1000 live births both in developing countries [2, 3] and in developed countries [4, 5]. However, the clinical manifestations of CP differ significantly between low- and high income countries [3, 6, 7], and some studies have reported that perinatal asphyxia and hyperbilirubinaemia are more common causes in developing countries [2, 3, 7] . In studies describing the panorama of CP, ascertainment of cases is a challenge even in developed countries [5]. In this regard, it is noteworthy that other studies in developing countries have reported CP prevalence between 3.4 and 4.5 per 1000 live births [3, 7].

Moldova is ranked as the country in Europe with the lowest gross domestic product (GDP) i per capita, estimated to around USD 5700 in 2017, compared with an average of USD 40000 for the European Union. Thus, financial resources for advanced medical care are limited, even if the proportion of the GDP spent for health expenditures (10%) is comparable to the proportion spent by the Nordic countries. These limited financial resources are a likely explanation of the high maternal mortality rate of 23 deaths per 100,000 live births; five to six times higher than in the Nordic countries, and of the infant mortality rate of 12 deaths per 1000 live births, being more than twice as high as in the Nordic countries (9). The prevalence of CP and distribution of CP subtypes, motor impairments and associated problems is unknown, and access to orthopaedic surgery as well as to modern treatment, such as Botulinum toxin injections are very limited (personal observation) [8, 9].

Thus, the aim of this study is to describe the panorama of CP in Moldova with emphasis on CP subtypes, the severity of the motor impairments and the occurrence of associated impairments, as well as how these clinical characteristics relate to perinatal risk factors.

## Methods

### Design and study population

Eligible for inclusion in this cross-sectional study were children with CP treated at the National Hospital Institute of Mother and Child, in Chisinau, Moldova, born between January 1st 2009 and December 31st 2010. This hospital is the reference hospital for children with disabilities living outside the Capital area, covering approximately 2.7 million (75%) of Moldova’s 3.5 mill inhabitants. Acquired CP lesions after the neonatal period were excluded. Children with neurological impairments are referred to this hospital every six months for examination and treatment.

Children diagnosed with CP were identified by the first author via medical records from the Departments of child neurology (3–18 years) and of toddler neurology (3 months to 3 years). The data were collected between June and September 2016, when the children were 7–8 years old. Detailed information regarding neurological status, motor function and associated impairments was identified by scrutinizing the medical records of each child from several departments. Antenatal and perinatal information was collected from the medical records at the Maternity ward, and at the Departments of neonatal care, premature birth and from the Neonatal Intensive Care Unit (NICU).

### Variables

CP was diagnosed and classified consistent with the recommendations proposed by the Surveillance of Cerebral Palsy in Europe (SCPE) [10] into spastic, dyskinetic, ataxic and not classified subtypes. The spastic subtype was sub-classified into unilateral and bilateral subtypes, spastic unilateral CP into right and left hemiplegia, and spastic bilateral CP into quadriplegia and diplegia in accordance with the International Classification of Disease 10 (ICD 10) [11].

Gross motor function was classified according to the Gross Motor Function Classification System (GMFCS) [12] using the available information on walking and sitting abilities. Fine motor function was classified according to the Bimanual Fine Motor Function (BFMF) scale using the available information on hand function [13]. As the data were collected from medical records of variable quality, the children’s gross motor function was categorized into those who were able to walk without assistive devices (corresponding to GMFCS level I and II), those who were able to walk with assistive devices corresponding to (GMFCS level III) and those who were unable to walk even with such devices (corresponding to GMFCS levels IV-V).

Fine motor function is described by speech therapists in the free text of medical records. Based upon these descriptions, fine motor function was classified according to BFMF, as proposed by Andersen et al. [4]. As for GMFCS, the BFMF classification was reduced to three categories; i.e. BFMF level I-II, BFMF level III and BFMF levels IV-V [4].

### Associated impairments

Speech therapists tested cognitive abilities in the hospital and described speech and feeding abilities in free text in the medical records.

*Cognitive* function was assessed using the Development Assessment of Young Children Evaluation tool (DAYC) and classified into intellectual disability (intelligence quotient (IQ) score &lt; 70) and normal intellectual ability (IQ score ≥ 70) [14].

*Speech* was classified on a five-level scale from zero to four, where zero indicated normal speech, level I indicated indistinct speech, level II obviously indistinct speech, level III severely indistinct speech (difficult to understand) and level IV indicated children with no speech.

*Feeding* ability was classified into five levels, 0 indicating that the child did not have feeding problems, level I indicating that the child was in need of some assistance, level II that the child was entirely dependent on assistance, level III that the child was partly tube fed and level IV that the child was mainly tube fed. No information on gastrostomy was recorded. The five levels were then dichotomised into two categories: independent feeding and dependent on caregiver assistance.

*Vision* was described as 0 for normal, 1 for impaired and 2 for severely impaired [10], and later dichotomised into 0 (0–1 normal or impaired) and 1 (2 severely impaired).

*Hearing* was described as 0 for normal, 1 for impaired and 2 for severely impaired, and later dichotomized into 0 (0–1 normal or impaired) and 1 (2 for severely impaired).

*Epilepsy* was defined as two unprovoked seizures, excluding neonatal and febrile seizures. Epilepsy was considered active if the child used antiepileptic drugs at the time of the registration.

### Perinatal data

Available perinatal data included gestational age (GA), birth weight (BW) and Apgar scores at one and five minutes. Gestational age at birth is usually estimated from ultrasonography at GA week 18–22. In this study, GA was categorized into extremely low (ELGA &lt; week 28, very low (VLGA: GA week 28 (28.0) to &lt; 32 (31 weeks; six days), moderately low (MLGA: GA week 32 (32.0) to 37 (36 weeks, six days) and term-born (GA week 37.0 or later). Birth weight was recorded to the nearest gram (g) and was categorized as extremely low (ELBW &lt; 1000 g); very low (VLBW: 1000–1499 g); moderately low (MLBW: 1500–2499 g) and normal (BW &gt; 2500 g). Apgar scores at one and five minutes were categorized into three categories: 0–3, 4–6 and 7–10.

### Statistical methods

The Statistical Package for Social Sciences (IBM SPSS V.19) was used for data analysis, and a significance level of 0.05 was chosen. Descriptive statistics with frequency tables were performed to show proportions of subtypes and CP severity levels, and the proportion of children with associated impairments. Differences in proportions were evaluated using Chi-square statistics, and *p*-values &lt; 0.05 were considered statistically significant.

### Ethics

The study was approved by the National Committee for Ethical Expertise in Clinical Trials (Nr. 266) and by the Centre of Early Intervention ‘Voinicel’ Ethical Committee (nr. 01/17). A statement of Agreement of Collaboration between the State Medical and Pharmacy University and the Institute of Mother and Child was signed, and finally, permission to conduct the study was obtained from the Ministry of Health (12th of July, nr. 08/88), from the Municipality Council of Social Protection, (13.07.2016, nr. 070117/957) and from the Ministry of Labour, Social Protection and Family of the Republic of Moldova.

Permission to access the children’s data was obtained from the Republican Ethics Committee, The State Medical and Pharmacy Ethics Committee, Ministry of Health and Ministry of Labour and Social Protection and the Directors of the Main Hospital for Children in RM- Institute of Mother and Child.

Parents or primary caregivers of patients treated at this centre sign an informed consent form stating that medical data collected as part of the admission may be used for research purposes. The use of this regularly collected medical information in the present study was approved as described above and did not require a new individual consent form to be completed.

## Results

In all, 207 children with CP were identified. The total number of live births during 2009 and 2010 in Moldova was 81,277, and a crude estimate of CP prevalence would be 3.4 per 1000 live births if the children in this study were recruited from 75% of the birth population. Of the 207 children, 22 had incomplete data. Thus, the study population comprised 185 children with a mean age of 7.3 years (range: 6.6–7.6 years) and 67 (36%) of these were girls. Thirty-seven children (20%) had spastic unilateral, 113 (61%) had spastic bilateral, 22 (12%) had dyskinetic and nine (5%) had ataxic CP. In four children (2%), the subtype could not be classified. Mean age at diagnosis was 23 months (range: 12-91 months). Among children with unilateral spastic CP, the extremities of the right side were affected in 21 (57%) and on the left side in 16 (43%) (*p* = 0.502). Among those with spastic bilateral CP, 19 (17%) had diplegic, while 94 (83%) had quadriplegic CP (*p* = 0.001).

Table 1 shows that 60 (31%) children were able to walk independently (GMFCS level I-II), 53 children (33%) were able to walk with assistive devices (GMFCS level III) and the remaining 72 (36%) children were unable to walk (GMFCS level IV-V) (*p* = 0.001). Table 1 also shows that 73 (40%) of the children had hand function corresponding to BFMF level I or II, 47 (24%) had BFMF level III and 65 (36%) had hand function corresponding to level IV or V (*p* = 0.001). Children with unilateral CP had better gross and fine motor function compared to children with bilateral and dyskinetic CP (*p* = 0.001) (Table 1).

**Table 1:** Subtypes, severity and associated impairments in a cohort of 185 children with CP

	CP subtypes						
	Unilateral N (%)	Bilateral N (%)	Dyskinetic N (%)	Ataxic N (%)	Not classified N (%)	Total N (%)	P-value of totals
GMFCS levels							
Level I-II	33 (89)	21 (19)	0	5 (56)	1 (25)	60 (33)	
Level III	4 (11)	39 (35)	4 (18)	4 (44)	2 (50)	53 (29)	
Level IV-V	0	53 (28)	18 (82)	0	1 (25)	72 (38)	&lt; 0.001
BFMF levels							
Level I-II	33 (91)	35 (31)	0	4 (44)	1 (25)	73 (40)	
Level III	4 (10)	32 (28)	4 (18)	5 (56)	2 (50)	47 (24)	
Level IV-V	0	46 (41)	18 (82)	0	1 (25)	65 (36)	&lt; 0.001
Intellectual disability							
No	31 (84)	39 (35)	2 (9)	3 (34)	1 (25)	76 (41)	
Yes	6 (16)	74 (66)	20 (91)	6 (67)	3 (75)	109 (59)	0.001
Active epilepsy							
No	29 (78)	48 (43)	8 (36)	3 (33)	4 (100)	92 (49)	
Yes	8 (22)	65 (58)	14 (64)	6 (67)	0	93 (51)	&lt; 0.001
Vision							
Normal/impaired	34 (92)	80 (71)	19 (86)	7 (78)	3 (75)	143 (77)	
Severely impaired	3 (8)	33 (29)	3 (14)	2 (22)	1 (25)	42 (23)	0.054
Hearing							
Normal/impaired	37 (100)	104 (92)	21 (96)	9 (100)	4 (100)	175 (95)	
Severely impaired	0	9 (8)	1 (5)	0	0	10 (5)	0.143
Speech impairments							
Normal	11 (30)	8 (7)	1 (5)	0	0	20 (11)	
Impaired, understandable	25 (68)	43 (38)	7 (32)	5 (56)	1 (25)	81 (44)	
Severely impaired/no speech	1 (3)	62 (55)	14 (64)	4 (44)	3 (75)	84 (45)	&lt; 0.001
Feeding							
Independent	34 (92)	56 (49)	8 (36)	5 (56)	2 (50)	105 (57)	
Dependent	3 (8)	57 (51)	14 (64)	4 (44)	2 (50)	80 (43)	&lt; 0.001
Total	37 (100)	113 (100)	22 (100)	9 (100)	4 (100)	185 (100)	

### Associated impairments

Table 1 shows that 109 (59%) children had an intellectual disability. In total, 42 (23%) had severe visual and ten (5%) severe hearing impairment, 93 (50%) had active epilepsy, 84 (45%) had severely impaired speech or no speech and 80 (43%) children were unable to eat independently (*p* = 0.001). Ten (5%) of the 185 children with CP were severely affected by all of these impairments. Table 1 also shows that associated impairments were most prevalent among children with dyskinetic and spastic bilateral CP subtypes (*p* = 0.001), while they were least prevalent among children with unilateral CP (*p* = 0.041). Among children with unilateral CP, there were no significant differences in the prevalence of associated impairments between children with right- or left-sided affection (*p* &gt; 0.056). All ten children who were severely affected by all associated impairments had the spastic bilateral subtype. None of the 84 children with severe speech impairment communicated with the help of augmentative or alternative communication aids (i.e. pictures or pictograms). The proportion of children without any associated impairments in this study was 11%.

### Perinatal risk factors

In total, 133 (72%) children with CP were born at term, whereas 10 (5%) were born ELGA. Among children born at term and preterm, the most common subtype was spastic bilateral CP, accounting for 54% and 79%, respectively (Table 2). Only one of 23 children born before GA week 32 was able to walk without assistive devices (GMFCS level I-II), compared to 11 (38%) children born moderately preterm, and 48 (36%) children born at term (*p* = 0.023). Five (22%) of the 23 children born before week 32 had good hand function (BFMF I-II); however, this proportion was lower when compared to those born at term, 54 (41%) of 133 children born ≥37 weeks had good hand function (*p* &lt;  0.001) (Table 3).

**Table 2:** Children with different CP subtypes by gestational age (GA), birth weight (BW) and Apgar score

	CP subtypes						
	Unilateral N (%)	Bilateral N (%)	Dyskinetic N (%)	Ataxic N (%)	Not classified N (%)	Total N (%)	*P*-value of totals
GA							
GA &lt; 28 weeks	1 (3)	7 (6)	1 (5)	0	1 (25)	10 (5)	
GA 28–31 weeks	0	11 (10)	0	1 (11)	0	12 (7)	
GA 32–36 weeks	4 (11)	23 (20)	2 (9)	1 (11)	0	30 (16)	
GA ≥37 weeks	32 (86)	72 (64)	19 (86)	7 (78)	3 (75)	133 (72)	0.002
BW							
BW &lt; 1000 g	0	6 (5)	0	0	0	6 (3)	
BW 1000-1499 g	1 (3)	8 (7)	1 (5)	0	1 (25)	11 (6)	
BW 1500-2499 g	5 (14)	27 (24)	4 (18)	1 (11)	1 (25)	38 (21)	
BW ≥2500 g	31 (83)	72 (64)	17 (77)	8 (89)	2 (50)	130 (70)	0.054
Apgar (1 min)							
Apgar 0–3	1 (3)	19 (17)	1 (5)	1 (11)	1 (25)	23 (12)	
Apgar 4–6	8 (22)	40 (35)	5 (23)	2 (22)	1 (25)	56 (30)	
Apgar 7–10	28 (75)	54 (48)	16 (72)	6 (67)	2 (50)	106 (58)	0.057
Apgar (5 min)							
Apgar 0–3	1 (3)	7 (6)	2 (9)	1 (11)	0	11 (6)	
Apgar 4–6	1 (3)	30 (27)	2 (9)	0	2 (50)	35 (19)	
Apgar 7–10	35 (94)	76 (67)	18 (82)	8 (89)	2 (50)	139 (75)	0.044
Total	37 (100)	113 (100)	22 (100)	9 (100)	4 (100)	185 (100)

**Table 3:** Severity of gross and fine motor impairments in children with CP by gestational age (GA), birth weight (BW) and Apgar score

Estimated GMFCS					Estimated BFMF			
	I-II N (%)	III N (%)	IV-V N (%)	Total N (%)	I-II N (%)	III N (%)	IV-V N (%)	Total N (%)
GA								
GA &lt; 28 weeks	1 (2)	5 (9)	5 (7)	11 (6)	4 (6)	3 (6)	4 (6)	11 (6)
GA 28–31 weeks	0	7 (13)	5 (7)	12 (7)	1 (1)	7 (15)	4 (6)	12 (7)
GA 32–36 weeks	11 (18)	11 (21)	7 (10)	29 (15)	14 (19)	9 (19)	6 (10)	29 (15)
GA &gt; 37 weeks	48 (80)	30 (57)	55 (76)	133 (72)	54 (74)	28 (60)	51 (78)	133 (72)
BW								
BW &lt; 1000 g	1 (2)	3 (6)	2 (3)	6 (3)	3 (4)	1 (1)	2 (3)	6 (3)
BW 1000-1499 g	2 (3)	4 (7)	5 (7)	11 (6)	3 (4)	4 (9)	4 (6)	11 (6)
BW 1500-2499 g	10 (17)	12 (23)	16 (22)	38 (21)	12 (17)	13 (28)	13 (20)	38 (21)
BW &gt; 2500 g	47 (78)	34 (64)	49 (68)	130 (70)	55 (75)	29 (62)	46 (71)	130 (70)
Apgar (1 min)								
Apgar 0–3	1 (1)	7 (13)	15 (21)	23 (13)	3 (5)	7 (15)	13 (20)	23 (13)
Apgar 4–6	16 (27)	16 (30)	24 (33)	56 (30)	20 (27)	16 (34)	20 (31)	56 (30)
Apgar 7–10	43 (72)	30 (57)	33 (46)	106 (57)	50 (68)	24 (51)	32 (49)	106 (57)
Apgar (5 min)								
Apgar 0–3	1 (2)	3 (5)	7 (10)	11 (6)	1 (2)	4 (9)	6 (9)	11 (6)
Apgar 4–6	6 (10)	11 (21)	18 (25)	35 (19)	9 (12)	10 (21)	16 (25)	35 (19)
Apgar 7–10	53 (88)	39 (74)	47 (65)	139 (75)	63 (86)	33 (70)	43 (66)	139 (75)
Total	60 (100)	53 (100)	72 (100)	185 (100)	73 (100)	47 (100)	65 (100)	185 (100)

*GMFCS* Gross Motor Function Classification System estimated from descriptions of walking and sitting abilities

*BFMF* Bimanual Fine Motor Function estimated from descriptions of hand function in each hand separately

*P-*value GMFCS/GA,BW,Apgar 1,5 min &lt; 0.002

Table 4 shows that epilepsy was present in a higher proportion of children born at term compared to those born preterm, while intellectual disability, severe vision and hearing impairments were more prevalent in children born before GA 28 weeks compared to those born at term (*p* = 0.024).

**Table 4:** Associated impairments by gestational age (GA) in children with CP

	GA &lt; 28 N (%)	GA 28–31 N (%)	GA 32–36 N (%)	GA &gt; 37 N (%)	Total N (%)	P-value of totals
Intellectual disability						
No (&gt; 70)	3 (30)	4 (33)	14 (47)	55 (41)	76 (41)	
Yes (&lt; 70)	7 (70)	8 (67)	16 (53)	78 (59)	109 (59)	0.553
Active epilepsy						
No	8 (80)	7 (58)	19 (63)	58 (44)	92 (49)	
Yes	2 (20)	5 (42)	11 (37)	75 (56)	93 (51)	0.008
Vision						
Normal/impaired	6 (60)	9 (75)	24 (80)	104 (78)	143 (77)	
Severely impaired	4 (40)	3 (25)	6 (20)	29 (22)	42 (23)	0.303
Hearing						
Normal/impaired	9 (90)	12 (100)	30 (100)	124 (93)	175 (95)	
Severely impaired	1 (10)	0	0	9 (7)	10 (5)	0.552
Speech Impairments						
Normal	1 (10)	0	4 (13)	15 (11)	20 (11)	
Impaired, understandable	4 (40)	7 (58)	15 (50)	55 (41)	81 (44)	
Severely impaired/No speech	5 (50)	5 (42)	11 (37)	63 (48)	84 (45)	0.994
Feeding						
Independent	7 (70)	6 (50)	20 (67)	72 (54)	105 (57)	
Dependent	3 (30)	6 (50)	10 (33)	61 (46)	80 (43)	0.347
Total	10 (100)	12 (100)	30 (100)	133 (100)	185 (100)	

*GA* Gestational Age (weeks) in categories (&lt; 28 weeks of gestation, 28–31 weeks, 32–36 weeks and &gt; 37 weeks of gestation)

A total of 130 (70%) children had normal BW, and six (3%) had BW &lt; 1000 g. Among children with normal BW, the most common CP subtype was spastic bilateral, 72 (64%) (*p* &lt;  0.001) (Table 2).

Finally, 79 (43%) children had a low Apgar score (0–3 and 4–6) at one and 46 (25%) at five minutes (Table 3). Low Apgar scores were associated with more severe impairments of gross and fine motor function (*p* = 0.029) and with spastic bilateral and dyskinetic CP (*p* = 0.057) (Table 3).

## Discussion

In this first study of the panorama of CP in Moldova we found a crude prevalence estimate of 3.4 per 1000 live births and that spastic bilateral CP was the most common subtype, accounting for nearly two-thirds of all cases in this population of children. Only every fifth child had the spastic unilateral CP subtype, and only one third could walk without an assistive device. Most of the children had significantly impaired fine motor function, and associated problems were common. Almost half of the children had barely understandable or no speech and a concern is that none of them used assisted or augmented communication. Only 28% of the children were born prematurely, and around 10% of the children were born at GA below 32 weeks, or with BW below 1500 g. Every fourth child had Apgar scores below 7 at five minutes.

### Validity

#### Strengths and limitations

The strength of the study is the hospital-based cohort from the main paediatric hospital of Moldova Institute of Mother and Child. As this hospital cares for all children living outside the Municipality of Chisinau, the cohort may be considered population-based and representative of 75% of the population of Moldova. The regular assessment of the children provided high-quality clinical information that could be systematised. The mean age of the children when the study was performed was 7.3 years old, which represents a strength regarding the validity of the CP diagnosis [9, 15]. Access to a variety of medical records also ensured ascertainment of the cases. Nonetheless, we cannot exclude that some children with milder forms of CP (for example, some children with unilateral spastic CP with GMFCS and BFMF level I), were not referred to this central hospital. Such selection bias could, therefore, have contributed somewhat to the low proportion of children with the unilateral CP subtype and the high proportion of children with severe motor impairments and associated problems. In contrast, the crude estimate of prevalence was high, and even in the most unlikely event that the cases included in this study would represent all children with CP in Moldova, the prevalence would be 2.5 per 1000 live births. The latter figure would still be high compared with other European countries [5, 16, 19].Thus, we consider it unlikely that the main findings and conclusions in this study can be completely explained by selection bias.

Another limitation is that the classification of gross and fine motor function (GMFCS and BFMF) was made in retrospect, based upon text descriptions in the medical records. However, the main criterion in the classification of GMFCS is the ability to walk with or without assistive devices or not at all. Therefore, we believe that misclassification of GMFCS is unlikely to explain the results regarding gross motor function. The classification of fine motor function (BFMF) according to Andersen et al. [4] is more complicated. Interestingly, the proportion of children with severely impaired fine motor function in this study is about the same as Andersen et al. [4]. However, it should be noted that fine motor function was found to be more impaired in Norway compared to other populations at that time. Thus, the findings for fine motor function in this study should be interpreted with caution. Assessments of cognitive abilities, speech and feeding were performed by experienced speech therapists, mostly using validated instruments, which is a strength of this study.

### Comparison with the literature

In the present study the distribution of spastic and non-spastic CP subtypes is comparable to other studies in Europe [4, 16].

However, the proportion of children with unilateral spastic CP is lower, while spastic bilateral (particularly quadriplegic CP) and dyskinetic forms were higher in our study compared to several studies in Europe and Australia [4, 16–18].

Among term born children, the proportion with dyskinetic CP in our study (12%) was lower compared with Himmelmann et al. (23%) [19], but higher than in two other studies [4, 19]. The percentage of children with the ataxic subtype is similar to other studies [4].

The distribution of CP subtypes among children born preterm in this study was comparable to the distribution reported by Himpens et al. [16] in their metanalysis of studies including children born later than 1980.

An important finding is that the proportion of children who were able to walk independently (33%) was lower in the present study than in a Norwegian study (55%), Andersen et al. [4], and in the majority of the studies included in the meta-analysis by Himpens et al. [16].

The proportion of children without any associated impairments was 11% in this study compared to 28% in Norway [4] and 48% in Sweden [19]. However, the latter study did not include speech impairments [19]. When we excluded speech impairments, we found that 35% of the Moldovan children with CP had no associated impairments (Table 4), which is comparable to results from Norway (34%) [4], but lower than in Sweden and Deutschland [19, 20].

The proportion of children with severely impaired or no speech in our study was higher than in a Norwegian study [4]. This finding is reasonable, considering the low proportion of children with unilateral spastic CP, and the high proportion of children with severe gross motor impairments (GMFCS level IV-V) in our study. However, it is concerning that none of the children with severely impaired speech had any form of assisted or alternative communication (ASC). This finding may be partially consistent with one study in Norway reporting that approximately 50% of children in need of such communication did not use ASC [21].

The proportions of children born prematurely, and born before week 28 were lower in Moldova than in two Nordic studies [4, 19]. This may be partly explained by lower proportions in the background population (0.27% in Moldova versus 0.4% in Norway) [22], but more likely by differences in perinatal and neonatal mortality. The majority of children with CP were born at term, consistent with most other studies.

Despite the higher proportions of children born at term and with quadriplegic and dyskinetic CP in this study, the proportion with low Apgar scores was similar to that reported by Andersen et al. [4] and Himmelmann et al. [19].

The most severe gross and fine motor impairments were associated with low Apgar scores. Moreover, children born with normal BW had more severe motor impairments in comparison with children with low BW (≤2500 g). These findings are consistent with another study conducted in Moldova between 2008 and 2014 [23].

#### Interpretation and implications

The lower proportion of children with unilateral CP in this study, as well as the lower proportion of children able to walk independently (GMFCS level I-II) could partly be explained by selection bias. It is possible that families living in the most rural areas of Moldova who have a child with mild unilateral CP are less likely to apply for or be referred to follow-up at the Institute of Mother and Child. However, if there are cases missing, the prevalence of CP must be significantly higher than our estimate. Thus, one implication of this study is that there is a need to establish national high-quality health registers in general, and CP registers in particular, in Moldova. This is of the utmost importance to provide health authorities with correct information regarding the prevalence and need for interventions but also to monitor ante- and perinatal health. Notably, a very recent Norwegian study documented the need for access to several health registers to ensure complete identification of cases [5].

However, although selection bias may partly explain the low proportion of children with unilateral CP, we consider it less likely to be the main explanation of the difference in CP subtypes between Moldova and other European studies. First, the crude estimate of prevalence is higher than in other studies, which makes it unlikely that there has been a significant underreporting of cases. Secondly, within the group of children with bilateral CP, the proportion with quadriplegic CP was very high, and finally, within the group with bilateral CP, the proportion able to walk independently was lower in our than in other populations, Thus, another interpretation may be related to obstetric and perinatal care. The increased maternal and infant mortality rates in Moldova compared to Norway, Sweden and Iceland, are most likely also reflected in higher perinatal and neonatal mortality rates. The high proportion of children with more severe manifestations of CP in Moldova may, therefore, also be explained by less advanced obstetric and neonatal care in Moldova than in Nordic countries. Both the spastic quadriplegic and dyskinetic CP subtypes are common in deliveries complicated by hypoxia-ischemia [24]. However, low Apgar scores were not more common in this study than in other studies. Therefore the higher proportion of children with quadriplegic and dyskinetic CP may have other causes, such as increased prevalence of pre-pregnancy or pregnancy related maternal disorders, congenital anomalies [25, 26], perinatal infections or placental disorders. The low proportion of children born before week 28 could, in part, be explained by different attitudes and limited access to advanced neonatal intensive care in Moldova. Thus, the other implication of this study is that there is a need to explore whether obstetric and neonatal care in Moldova may need to be further improved to achieve results comparable to other European countries.

Finally, this study has revealed that standard classification systems like GMFCS and BFMF, as well as motor function assessment tools, such as Gross Motor Function Measurement (version 66 or 88) [12], are not clinically used on a regular basis. Moreover, assisted or augmented communication was apparently not used by children with speech problem. One implication of these results will, therefore, be to establish a collaborative network with specialists from the rehabilitation centres throughout the country in order to improve the quality of care for children with CP in Moldova.

## Conclusion

Compared to other populations, children with CP in Moldova more frequently present with bilateral and dyskinetic subtypes. In addition, severe gross motor impairments and associated problems are also more common in Moldova. Our findings suggest a higher prevalence of CP, and the causal pathways may be different in Moldova relative to other European countries. Children with milder CP symptoms may be less likely to receive specialist care. These findings suggest a need for a national health quality register [27], and provide a basis to improve the quality of perinatal medicine and care for children with CP.

## Abbreviations

BFMF: Bimanual fine motor function
BW: Birth weight
CP: Cerebral palsy
GA: Gestational age
GDP: Gross domestic product
GMFCS: Gross motor function classification system
IQ: Intellectual quotient
SCPE: Surveillance of Cerebral Palsy in Europe
USD: United States dollars
